# Diabetes and climate change: current evidence and implications for people with diabetes, clinicians and policy stakeholders

**DOI:** 10.1007/s00125-023-05901-y

**Published:** 2023-03-25

**Authors:** Jacqueline M. Ratter-Rieck, Michael Roden, Christian Herder

**Affiliations:** 1 Institute for Clinical Diabetology, German Diabetes Center, Leibniz Center for Diabetes Research at Heinrich-Heine-University Düsseldorf, Düsseldorf, Germany; 2 German Center for Diabetes Research, Partner Düsseldorf, München-Neuherberg, Germany; 3 Department of Endocrinology and Diabetology, Medical Faculty and University Hospital Düsseldorf, Heinrich-Heine-University Düsseldorf, Düsseldorf, Germany

**Keywords:** Air pollution, Climate change, Complications, Heat, Physico-chemical environment, Planetary health, Review, Virus

## Abstract

Climate change will be a major challenge for the world’s health systems in the coming decades. Elevated temperatures and increasing frequencies of heat waves, wildfires, heavy precipitation and other weather extremes can affect health in many ways, especially if chronic diseases are already present. Impaired responses to heat stress, including compromised vasodilation and sweating, diabetes-related comorbidities, insulin resistance and chronic low-grade inflammation make people with diabetes particularly vulnerable to environmental risk factors, such as extreme weather events and air pollution. Additionally, multiple pathogens show an increased rate of transmission under conditions of climate change and people with diabetes have an altered immune system, which increases the risk for a worse course of infectious diseases. In this review, we summarise recent studies on the impact of climate-change-associated risk for people with diabetes and discuss which individuals may be specifically prone to these risk conditions due to their clinical features. Knowledge of such high-risk groups will help to develop and implement tailored prevention and management strategies to mitigate the detrimental effect of climate change on the health of people with diabetes.

**Graphical abstract:**

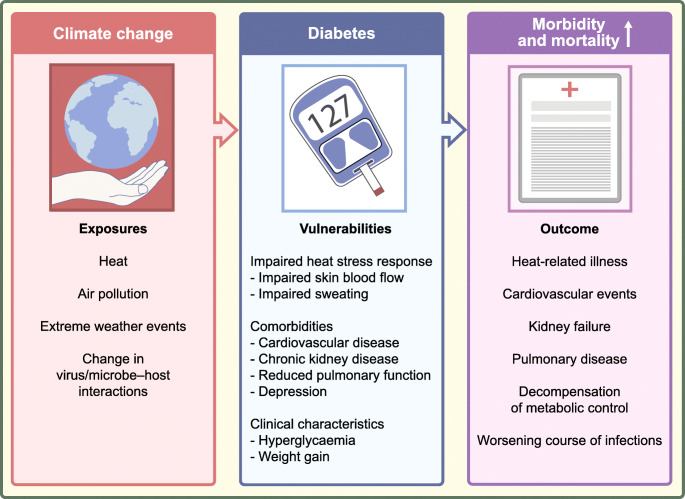

**Supplementary Information:**

The online version contains a slideset of the figures for download available at 10.1007/s00125-023-05901-y.

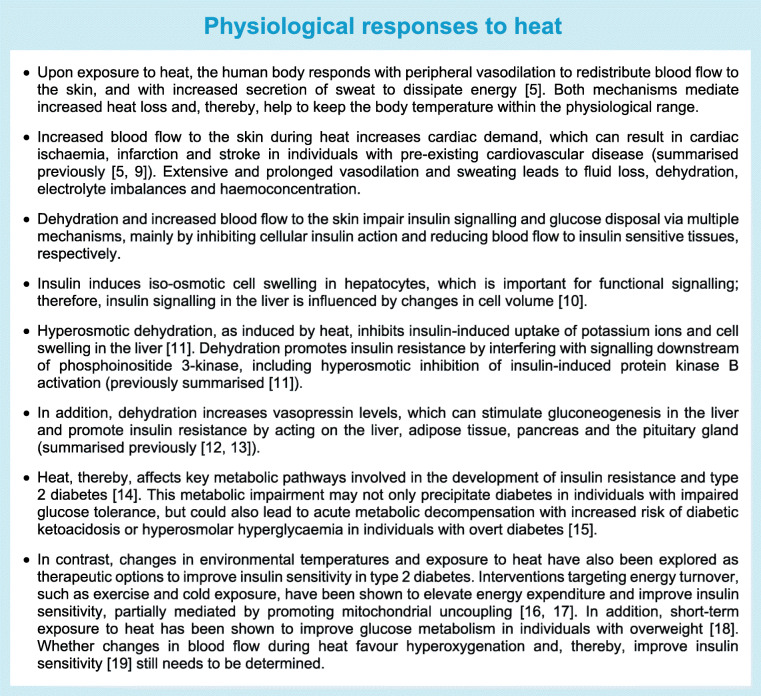

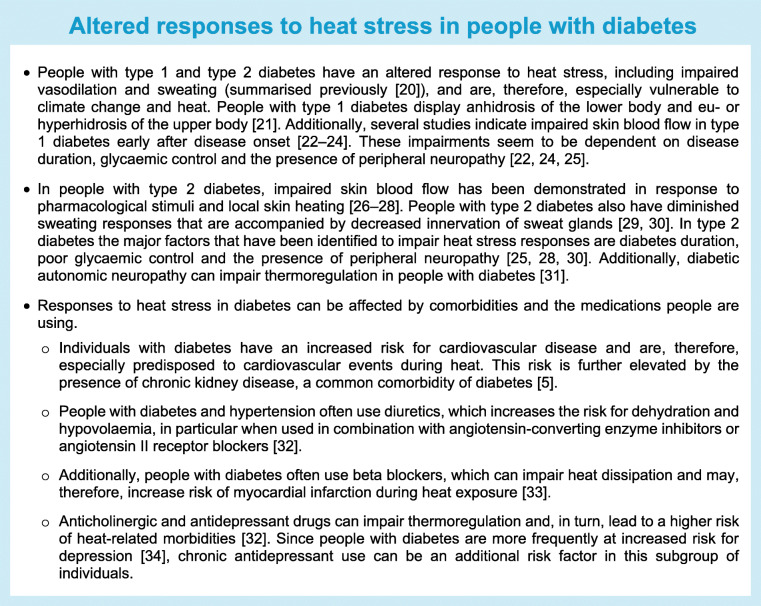

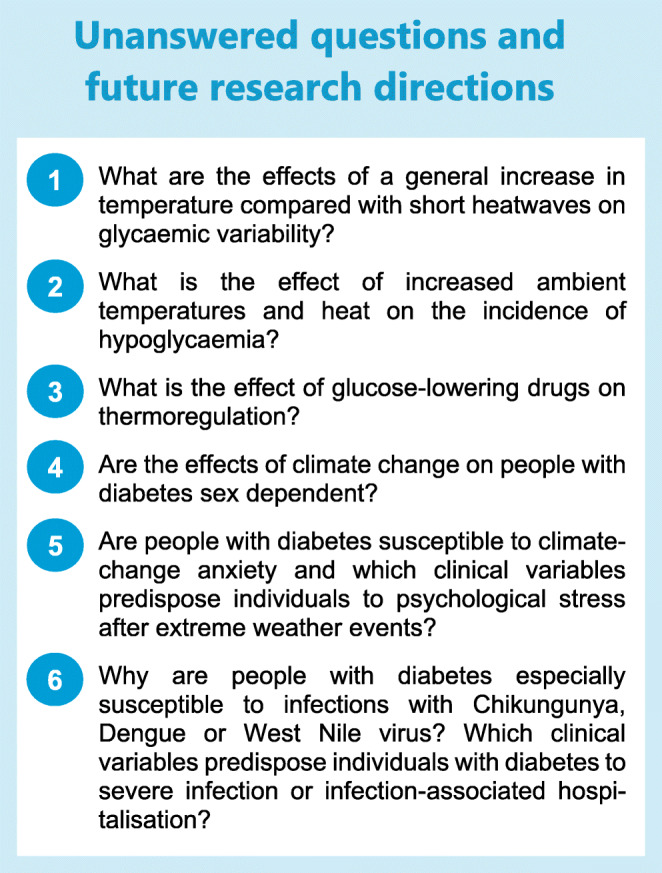







## Introduction

Climate change is a threat for planetary and human health. One of the most dangerous consequences of global warming is extreme heat, with over 345,000 heat-related deaths worldwide in people older than 65 years in 2019; this is 80.6% higher than the average in 2000–2005 [1]. Heatwaves, such as those in the Pacific Northwest in 2021 or in Europe in 2022, are not only breaking records of extreme temperatures, but are also likely to occur much more frequently in the near future [2–4]. Under high emission scenarios, temperatures above 40°C may, for example, occur in the UK every 3.5 years, whereas they were estimated to return every 100–300 years in the present climate [4].

Many factors can affect the physiological response to heat (see Text box: Physiological responses to heat). Among the individuals most vulnerable to extreme heat are infants and people older than 65 years, people living in urban environments and individuals with chronic diseases [5]. Both factors of the external (e.g. built, social and physico-chemical environment) and internal (e.g. proteome, microbiome, metabolome) exposome [6] will, therefore, determine vulnerability to climate change. Partially owing to impaired responses to heat stress, but also because of comorbidities like cardiovascular disease and chronic kidney disease, people with diabetes are particularly susceptible to the risks of high ambient temperatures and heatwaves (see Text box: Altered responses to heat stress in people with diabetes). This is concerning because the number of days that people are exposed to extreme heat has increased over recent decades [7], while, in parallel, the number of people living with diabetes reached 536 million in 2021 and is expected to increase to 783 million in 2045 [8].

In this review, we will provide an overview of studies looking into the influence of environmental factors associated with climate change on people with diabetes. In particular, we will focus on heat, air pollution, extreme weather events and infectious diseases that are expected to affect more individuals with global warming. For this purpose, a literature search in PubMed was performed, which included the search terms ‘diabetes’, ‘climate change’, ‘heat’, ‘ambient temperature’, ‘air pollution’, ‘wildfire’, ‘extreme weather event’, ‘Chikungunya virus’, ‘West Nile virus’ and ‘Dengue virus’. Reference lists of identified articles and articles citing the identified articles were screened to check for further potential studies for inclusion. Subsequently, we also suggest potential mitigation strategies to reduce the impact of climate change on the health of people with diabetes.

## Heat

### Associations of temperature with incidence of diabetes and glycaemic control

Currently, only few studies have investigated the relationship between ambient temperature and diabetes. Blauw et al analysed the association of changes in outdoor temperature and incidence of diabetes in the USA between 1996 and 2009 [35]. After adjustment for the prevalence of obesity, this ecological study found an increase in diabetes incidence (diagnosis of type 1 or type 2 diabetes) with higher temperatures, and the authors suggested that a 1°C rise in outdoor temperature may be associated with over 100,000 new diabetes cases per year in the USA. Since BMI data were categorical and only country- or state-level aggregated data were used, further studies using individual-level data and assessing BMI as a continuous variable are warranted.

Analysing worldwide data for the prevalence of diabetes, as diagnosed by fasting blood glucose ≥7.0 mmol/l or medication for elevated blood glucose levels, the same authors found an association between mean annual temperature and country-wise age-, sex-, income- and obesity-adjusted prevalence of increased fasting blood glucose levels [35]. In accordance, ambient temperature was associated with the prevalence of dysglycaemia and insulin resistance in a cohort of Spanish adults [36]. The increase in fasting blood glucose levels and insulin resistance with increased ambient temperatures may be partially mediated by differences in type and frequency of physical activity, which varies with different seasons and environmental temperatures [37]. Nevertheless, model adjustment for physical activity only modestly decreased the association of ambient temperature with insulin resistance [36].

In addition, although analysis of food frequency questionnaires did not suggest an association of ambient temperature with food intake [36], future studies should investigate the impact of the consumption of sweetened beverages, accessibility to fresh food and heat-associated psychological stress on the relationship of ambient temperatures with insulin resistance. Physiological mechanisms that mediate the association between increased ambient temperatures and insulin resistance may be activated by dehydration and its subsequent impact on insulin resistance and hepatic gluconeogenesis [11, 12], as well as temperature changes and their impact on brown adipose tissue activity (which is found to increase with cold temperatures) [38, 39].

In contrast, findings from a recent study suggest that 10 days of passive heat acclimation at approximately 34°C for 4–6 h per day improved glucose metabolism and increased fat oxidation in overweight individuals without affecting insulin sensitivity [18]. Physiological adaptations following these short-term increases in temperature will likely differ to those following long-term exposures to extreme temperatures in a heatwave. Moreover, environmental conditions in experimental climate chambers do not fully represent natural changes in ambient temperature, which are accompanied by, for instance, alterations in humidity and air pollution.

### Associations of temperature with hospitalisation and mortality risk in people with diabetes

Dysregulated blood glucose levels with increased ambient temperatures may increase the need for medical advice in individuals with diabetes. An analysis of 4,474,943 consultations of general practitioners in a cohort of people with type 2 diabetes in England from 2012–2014 showed an increased OR (1.097 [95% CI 1.041, 1.156]) per 1°C increase in temperature above 22°C for people seeking medical advice [40]. People with diabetes and additional cardiovascular comorbidities and those who were older than 65 years were particularly at risk. Although higher ORs were found in these groups, interaction terms were not significant because of limited statistical power.

A study investigating the impact of the July 1995 heat wave in Chicago (IL, USA) on excess hospital admissions revealed 11% more admissions in contrast to comparison weeks (average of 1 week before the heatwave and 3 weeks from the previous year without a heatwave), including a significant increase in admissions due to heat-related diagnoses. Although there was no increase in admissions owing to diabetes as a primary health condition for which the individual was hospitalised, 30% of people among the excess admissions had underlying diabetes, with a significant increase in admissions of people with non-insulin-dependent diabetes [41]. In contrast, a study analysing the effect of the 2006 heat wave in California, USA found an elevated risk ratio for emergency department visits (for individuals of white ethnicity), but not hospitalisations for diabetes [42]. A study analysing emergency room visits in California in the warm seasons of 2005–2008 found a positive association between same-day apparent temperature and admissions due to diabetes diagnosis [43], and a nationwide case-crossover study in Brazil showed that hospitalisations associated with diabetes increased by 5% for every 5°C increase in daily mean temperature [44]. Stratification for diabetes type (type 1, type 2, malnutrition-related, other specified or unspecified) did not show specific differences between the groups. However, older people, especially those older than 80 years, were particularly prone to hospitalisation. Along the same lines, a study of hospital admission records in Michigan, USA showed that people with diabetes who were older than 65 years had a higher risk of dying on hot days than people without diabetes (OR 1.11 [95% CI 1.04, 1.32]) [45]. An important risk factor here may be cardiovascular disease, since a higher risk for hospital admissions due to acute myocardial infarction was observed in individuals with diabetes when temperatures were above 28.8°C in the hot season in Hong Kong [46].

Taken together, several studies suggest an association between heat waves and a greater number of hospitalisations of people with diabetes. It remains to be determined to what extent these effects can be directly attributed to increased temperatures, and also to lifestyle factors that may be altered during periods of elevated temperatures, such as reduced physical activity or altered nutrition. In addition, long-term adaptations to hot environments at low latitudes, and increased prevalence of diabetes in studies comparing data over different years may also partially contribute to the association between heat waves and increased hospitalisation of people with diabetes. Mechanistically, it remains to be determined whether temperature-induced changes in heat shock proteins or brown adipose tissue metabolism play a contributory role [47].

Of note, in addition to heat extremes, cold temperatures have also been shown to influence specific risks among people with diabetes. A time-series analysis of people with diabetes in Taiwan revealed an inverse relationship between ambient temperature and admission rates for diabetic ketoacidosis and hyperglycaemic hyperosmolar state [48], and a recent study in Korea found an association of exposure to cold spells with increased risks for hospital admission and mortality due to diabetes [49]. Along the same lines, a study analysing mortality rates of diabetes and kidney disease in individuals from six different countries suggested that cold was an even greater burden than heat [50]. In addition, a study analysing daily deaths owing to diabetes in four cities in the Philippines detected elevated mortality risk both at low and high ambient air temperatures [51]. In conclusion, both hot and cold non-optimal temperatures may affect the burden of diabetes.

## Air pollution

The association of climate change and air pollution is characterised by complex interactions. On the one hand, increased production of industrial emissions like CO_2_, particulate matter and greenhouse gases, which are also increased by agriculture and land use, plays a central role in climate change and increases global warming. On the other hand, climate-change-induced meteorological alterations in temperatures and precipitation can impair air quality by increasing the levels of particulate matter and ground-level ozone [52]. Of note, studies reported a two-way interaction of air pollution and temperature on daily rates of all-cause and cardiovascular disease-associated mortality, with higher effects of air pollution on days with high air temperatures [53].

Multiple studies have shown that air pollution is associated with insulin resistance and an increased risk of developing diabetes [54, 55], and it has been estimated that approximately one-fifth of the global burden of type 2 diabetes may be attributable to pollution owing to fine particulate matter <2.5 μm (PM_2.5_) [56]. Additionally, air pollution contributes to diabetic complications. Several studies have indicated that, for instance, PM_2.5_ and NO_2_ increase risk of cardiovascular disease-associated mortality, and increase incidence of myocardial infarction and heart failure (reviewed previously [9]). Additionally, both particulate and gaseous pollutants have been associated with prevalent and incident distal sensorimotor polyneuropathy (DSPN) in people with obesity, but not in those without obesity [57]. Overall, the expected increase in air pollution in the future will likely contribute to the worldwide increase in the burden of diabetes, as well as the frequency of complications.

Besides these long-term effects, short-term effects of air pollution on diabetes are relevant, for instance, during wildfires. Wildfire smoke contains multiple air pollutants, including particulate matter, ozone and carbon monoxide. PM_2.5_ is a major air pollutant in wildfire smoke and increases significantly during wildfires; for instance, in Canada, during the 2015 wildfire event in the Pacific Northwest, PM_2.5_ increased from 6.8 μg/m^3^ to 52.3 μg/m^3^ [58]. Increasing ambient temperatures and periods of drought are causing the number and extent of wildfires to increase worldwide; during 2017–2020 compared with 2001–2004, the exposure to wildfires increased in 134 out of 185 countries [1]. Since wildfires can expose large regions to smoke [59], they may affect many people with diabetes. Overall, increasing concentrations of PM_2.5_ during and after a wildfire were found to be associated with more physician visits for respiratory, but not cardiovascular diseases in the general population [58]. A subgroup analysis of individuals with pre-existing diabetes revealed an increased risk of all-cause respiratory- and cardiovascular-related physician visits after wildfires, and an increased risk of cardiovascular morbidity, particularly in people with diabetes older than 65 years. In line with this, the level of PM_2.5_ during the 2017 wildfire in the North San Francisco Bay (CA, USA) was associated with emergency department visits for asthma and hospitalisations for respiratory disease, chronic lower respiratory disease and diabetes [60]. Reduced pulmonary function is a frequent comorbidity in type 2 diabetes [61, 62] and may be a major determinant of increased respiratory-related physician visits in individuals with diabetes. Moreover, obesity may be a risk factor for respiratory disease after exposure to wildfire smoke [63]. Whether wildfires are also associated with impaired glycaemic control, as suggested by the negative effects of short-term PM_2.5_ exposure on fasting blood glucose levels [64], is currently unknown. Furthermore, it will be important to determine the extent to which the effects of wildfires on blood glucose levels or hospitalisation of people with diabetes are directly attributable to short-term increases in air pollution and how other factors, such as psychological stress or the accessibility of healthcare services, may mediate and modify the morbidities described above.

## Extreme weather events

The intensity and frequency of weather extremes have increased over recent decades and can be largely attributed to anthropogenic climate change [65, 66]. Rising numbers of heatwaves, droughts, heavy precipitation, flooding and storms are a severe risk for public health and expose people with diabetes to multiple challenges including impaired access to primary care, hospitals and medication, as well as increased psychological stress and alterations in lifestyle that affect glycaemic control and diabetes management. Although the actual impact of extreme weather events on people with diabetes may be very heterogeneous due to local differences in the extent of their effects on medical infrastructure, local-disaster management and differences in healthcare systems, multiple studies suggest that people with diabetes may require specific prevention and control strategies in the context of weather extremes.

Several studies indicate that natural disasters may impair glycaemic control. A study from England, UK showed that people with diabetes who were affected by the floods in Hull and East Yorkshire in 2007 had higher mean HbA_1c_ levels 12 months after the floods compared with 12 months before the floods in contrast to people with diabetes who were not affected by the floods [67]. The main effect was attributed to people treated with insulin; in this group, HbA_1c_ levels were especially high at 6–9 months after the event and were normalised by 12 months following the floods. A higher risk for impaired glycaemic control after natural disasters in people with insulin-dependent diabetes is also suggested by a study demonstrating a significant association between fasting C-peptide levels and worsening glycaemic control 1 month and 3 months after the 2011 Tohoku earthquake and tsunami in Japan [68]. Although the number of earthquakes is not related to climate change, short- or long-term consequences on the health status of people with diabetes may be comparable to those after climate-change-associated extreme weather events. In a study of people affected by Hurricane Katrina in New Orleans (LA, USA) in 2005, worsened glycaemic control was observed as late as 16 months after the event [69], indicating long-term effects of such disruptions. Interestingly, the rise in HbA_1c_ levels differed between study participants using private and state-funded healthcare systems, possibly because the healthcare services differed in availability of diabetes-specific specialty clinics. This suggests an effect of socioeconomic disparities on glucose management after the extreme weather event. Such health disparities may not only be increased by natural disasters, but may also be reinforced by crises, such as the COVID-19 pandemic [70, 71], and highlight the need for targeted interventions, particularly after extreme weather events. In addition to glycaemic control, blood pressure and LDL-cholesterol levels deteriorated in some groups 16 months after the event [69]. Thus, extreme weather events may also elevate the risk for the development of diabetes-related complications.

Changes in HbA_1c_ levels or blood pressure may also increase the risk for hospitalisations of people with diabetes. People with diabetes older than 65 years visited the emergency department more often compared with other vulnerable populations 1 week after Hurricane Sandy [72]. Individuals visiting the emergency department and having a secondary diagnosis of diabetes often presented with hypertension, myocardial infarction or hypertensive kidney disease. A long-term follow-up analysis even showed that senior citizens with diabetes who resided in counties that were highly impacted by Hurricanes Katrina and Rita not only had higher all-cause mortality risk directly after the incident, but also up to 10 years later [73].

Since depression is a frequent comorbidity of diabetes [34], people with diabetes may also suffer from an increased psychological burden after natural disasters. Although the presence of diabetes was not associated with mental-health distress after the earthquake/tsunami/nuclear disaster in Japan 2011, there was an association of suboptimal glycaemic control (HbA_1c_ ≥7% [53 mmol/mol]) with psychological distress and possible post-traumatic stress disorder [74]. Consequently, people with diabetes may require additional psychological support after extreme weather events.

## Infections

Climate-change-associated changes in temperature, humidity and precipitation may affect the transmission of infectious diseases. It was recently estimated that over 58% of infectious diseases, including viral, bacterial and fungal infections, could be aggravated by climate change [75]. Although it is difficult to isolate the role of climate change in driving transmission of infectious diseases, multiple studies suggest that rising temperatures and droughts will contribute to the global spread of several vector-borne infectious diseases. Rising temperatures have already facilitated the expansion of the Asian tiger mosquito (*Aedes albopictus*) in Europe, which transmits vector-borne diseases such as those caused by Zika, Dengue and Chikungunya virus [76–78]. Additionally, heat waves and droughts also likely contribute to epidemics of West Nile fever [77, 79]. Although the transmission of vector-borne diseases is not only determined by climate, but is also influenced by sociodemographics, public-health systems, globalisation and other environmental factors [76], it is important to consider whether people with diabetes may be at risk for future epidemics; this is particularly important since it is well established that people with diabetes have an elevated risk for severe infections and hospital admissions for several infectious diseases [80]. This can be partially explained by alterations in immune-cell frequencies [81] and immune responses [82] in individuals with diabetes.

In individuals infected with Chikungunya virus, diabetes is the second most prevalent comorbidity after hypertension; of note, the prevalence of diabetes was higher in infected individuals older than 50 years compared with younger individuals [83]. Moreover, people with diabetes had an increased risk for developing severe disease after infection, which is characterised by rheumatological and neurological symptoms that can persist for several months or years after acute infection. Similarly, several studies have shown that, in addition to age and hypertension, diabetes is a risk factor for the development of encephalitis or death after West Nile virus infection [84, 85]. Murine studies that used diabetes models have shown that delayed immune responses and reduced leucocyte infiltration into the brain may contribute to an increased severity of West Nile virus infection in diabetes [86, 87]. A recent experimental study in female mice also identified obesity as a risk factor that increases the disease severity of West Nile virus infection [88]. Having diabetes or diabetes-related hypertension also increased the risk of dengue haemorrhagic fever (DHF) in a Chinese population [89]. DHF is a severe form of dengue fever and is characterised by bleeding and plasma leakage. Diabetes was confirmed as a risk factor for dengue fever in a Brazilian population [90] and, furthermore, more severe thrombocytopaenia was found during Dengue virus infections in people with diabetes compared with those without [91]. This likely contributes to an increased risk for admissions to intensive care units for people with diabetes and dengue fever [92].

Although the current literature suggests an increased risk for a more severe course of disease in people with diabetes after infection with Chikungunya, West Nile or Dengue virus, the molecular mechanisms underlying these associations remain elusive. Additionally, people with diabetes may also have an increased risk for a severe course of disease with several other infections that may spread increasingly as a result of climate change [75] but that could not be discussed in detail here. Future studies investigating immune responses in infected people with diabetes compared with healthy individuals will, therefore, be of great importance and may help to identify subgroups with an increased risk for severe disease. Importantly, immune responses may also be affected by alterations in the microbiome–immunity axis as a result of the effects of climate change on human diets [93].

## Management and mitigation strategies

**Fig. 1:**
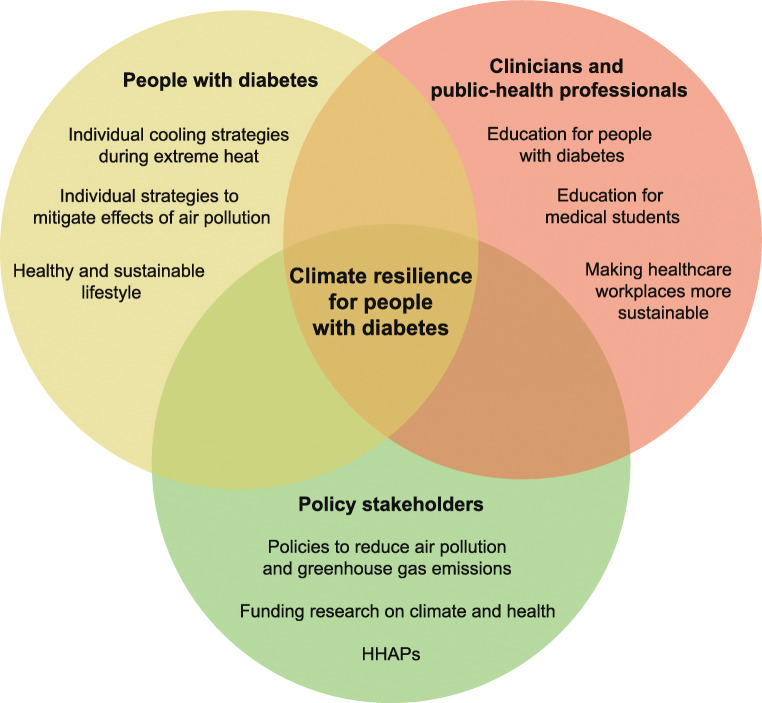
An overview of climate change adaptation and mitigation strategies for people with diabetes, clinicians, public-health professionals and policy stakeholders. People with diabetes, clinicians, public-health professionals and policy stakeholders can mitigate the effects of climate change by reducing greenhouse gas emissions at an individual and national level. Moreover, people with diabetes can apply individual adaptation strategies to reduce the effects of air pollution and extreme heat on personal health. Clinicians and public-health professionals can support people with diabetes by providing education and policy stakeholders can ensure timely public measures by implementing HHAPs. This figure is available as part of a downloadable slideset

Taken together, heat, air pollution, extreme weather events and infections all represent severe climate-change-associated health threats for people with diabetes. In order to mitigate their effects on morbidity and mortality, and to limit the associated healthcare costs, a joint effort by multiple stakeholders, including people with diabetes, clinicians, public-health professionals and politicians, is required (Fig. 1). Individual adaptation measures and policy strategies may not only mitigate the negative effects of climate change on human health, but could also represent an opportunity for a healthy and sustainable future.

Individuals with diabetes can apply personal strategies to minimise the effects of heat and air pollution on health. Dependent on the individual’s environment and resources, different cooling strategies, including the use of electric fans or reducing physical activity, can diminish the effects of heat [94]. Additionally, limiting outdoor activities and wearing face masks are personal-level strategies that can be applied to reduce exposure to and health effects of high air pollution [95]. Structured and easy-to-apply information on the respective strategies can be valuable resources and are already offered on multiple platforms for people with diabetes, including diabinfo [96], Diabetes UK [97] and the Centers for Disease Control and Prevention [98].

Moreover, clinicians can support individuals with diabetes when selecting appropriate personal mitigation strategies and provide them with relevant information. Medications prescribed to people with diabetes can affect the heat response [32] and, therefore, clinicians can advise on whether adjusting medications, e.g. reducing the dose of diuretics during heat waves, could be beneficial for patients under specific circumstances. Additionally, people with insulin-dependent diabetes should be advised by medical personnel about the stability of insulin and the potential of altered absorption during elevated temperatures [99], as well as the impact of heat on the shelf life of other glucose-lowering and diabetes-related medications. Studies indicate that a large number of health professionals recognise the impact of climate change on human health, but often time constraints are a barrier for engaging in communication [100]. Therefore, professional societies could support diabetologists by providing useful resources, including patient education materials on the risks of heat and air pollution, for example. Additionally, initiatives like the Green Diabetes Initiative of the Diabetes Technology Society could help to develop guidance for more sustainable waste management in daily practice [101]. To provide clinicians with sufficient knowledge on the impact of climate change on vulnerable groups, planetary-health education should be included in curricula for medical students [102] and, during their specialisation, medical fellows should be trained on how to integrate this knowledge in the treatment of individuals with diabetes in their daily practice.

The individual-level behaviour of people with diabetes and clinicians with regard to climate change mitigation strategies can be limited or supported by national or international policies. Worldwide, 190 countries have signed the Paris agreement, indicating their will to take action in order to limit the extent of global warming. Yet, to reach, or at least get close to the ambitious goals and limit the impact of anthropogenic climate change on human health, efforts to reduce global greenhouse gas emissions have to increase rapidly [103]. Furthermore, in order to reduce effects of heat and air pollution, mitigation strategies have to be developed and implemented. Heat-health action plans (HHAPs) could be a core element of national prevention and control strategies. A recent study indicated that 17 out of 27 countries analysed in the European region already have a national HHAP in place and have at least partially implemented five out of eight HHAP core elements [104]. Considering the needs of people with diabetes, it is encouraging that the core element ‘particular care for vulnerable groups’ has already been implemented, at least partially, by all countries, and that the majority of HHAPs specifically address people with chronic conditions [104]. Nevertheless, a lack of implementation of the core elements ‘preparedness of health and social systems’ and ‘long-term urban planning’ reveals that there is still great potential for governments to mitigate the effects of climate change on human health [104]. Collections of scientific studies on relevant intervention strategies can form the groundwork for implementation of blue and green infrastructure in urban planning [94], as well as implementation of measures to reduce air pollution and associated health effects [105].

## Conclusions and future perspectives

**Fig. 2:**
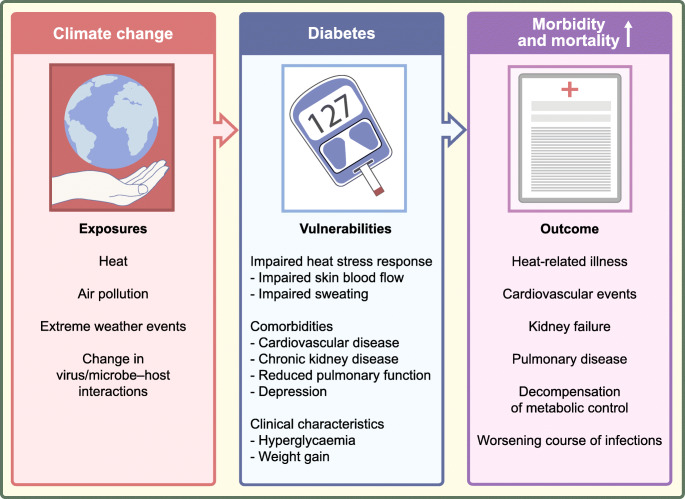
An overview of climate-change-associated health risks and consequences for people with diabetes. Climate change is associated with extreme heat, increased air pollution, extreme weather events and changes in virus and microbe–host interactions. An impaired heat stress response, diabetes-associated comorbidities and specific clinical characteristics make people with diabetes particularly vulnerable to climate-change-associated environmental changes, resulting in increased morbidity and mortality. This figure is available as part of a downloadable slideset

People with diabetes are particularly vulnerable to climate-change-associated health risks like heat, air pollution, weather extremes and specific infectious diseases (Fig. 2). In order to foster climate resilience, we need to enforce sustainable medical research, prevention and control strategies.

Although several studies indicate an increased burden of climate change on people with diabetes, many questions remain to be answered in the future (Text box: Unanswered questions and future research directions). More studies to identify additional predisposing clinical factors (Table 1) will be important, helping us to develop and implement prevention strategies, such as education of patients and physicians to increase the awareness of climate-change-associated risks, as well as organising (inter)national systems for risk assessments and heat warnings, which can be provided by national meteorological services to inform the general public and public-health authorities in a timely manner [106]. In addition to prevention strategies, action plans to support individuals with diabetes during periods of heatwaves or extreme weather events can help to reduce dysglycaemia and other diseases among the growing population of people with diabetes.

**Table 1:** Clinical characteristics of individuals with diabetes and expected implications for climate-change-associated risks

Clinical variable	Expected implications for climate-change-associated risks	References
Older age	Risk for hospitalisation and mortality during heat ↑	[5, 40, 45]
	Risk for severe disease after West Nile virus infection ↑	[84, 85]
	Risk for cardiovascular morbidity in the context of wildfires ↑	[58]
Obesity	Risk for DSPN with higher air pollution ↑	[57]
	Risk for severe disease after West Nile virus infection ↑	[88]
High HbA_1c_	Psychological distress after extreme weather events ↑	[74]
Insulin use	Glycaemic variability and risk of hypoglycaemia during heat ↑	[15]
	Glycaemic variability after extreme weather events ↑	[67]
Hypertension	Risk for severe disease after Chikungunya, West Nile or Dengue virus infection ↑	[83–85, 89]
Cardiovascular disease	Risk for heat-associated mortality ↑	[5, 40, 46]
Chronic kidney disease	Risk for heat-associated mortality ↑	[5]

DSPN, distal sensorimotor polyneuropathy

Because of differences in external and internal exposomes, not all people with diabetes will be similarly vulnerable to risks associated with heat, air pollution, extreme weather events or infectious diseases. Overall, old people with diabetes and those with comorbidities are most likely to be affected by climate-change-related health risks. In light of the recent approaches for diabetes reclassification [107–109], it is tempting to speculate that because of differences in clinical variables and comorbidities, some diabetes endotypes may be more vulnerable to climate-associated factors, such as heat. High-risk phenotypes, for instance people with severe insulin-resistant diabetes (SIRD), may be especially vulnerable to heat due to frequent comorbidities, like hypertension and cardiovascular disease [110, 111], but may also have a different risk of developing infections because of their altered immune system [112, 113]. Future studies investigating diabetes heterogeneity may, therefore, help to identify specific climate-change-associated risks for different patient groups.

In addition to mitigation strategies for individuals with diabetes, strategies to prevent diabetes are of great importance to limit further expansion of the population of risks. Of note, the relationship between environmental factors and health is reciprocal and, therefore, prevention strategies, such as sustainable diets, may not only improve health, as assessed by all-cause and cardiovascular mortality, but also reduce the environmental footprint, thereby fostering planetary health [114].

## Supplementary information

ESM 1 (PPTX 292 kb)

## Abbreviations

DHF: Dengue haemorrhagic fever
HHAP: Heat-health action plan
PM_2.5_: Fine particulate matter <2.5μm
