# Supplementary material for: Diabetes and climate change: current evidence and implications for people with diabetes, clinicians and policy stakeholders
Source: Diabetologia. 2023 Mar 25;66(6):1003–15. doi: 10.1007/s00125-023-05901-y (PMC10039694; doi:10.1007/s00125-023-05901-y)
Supplement: Supplementary file 1 — (PPTX 292 kb) [file 125_2023_5901_MOESM1_ESM.pptx]

## Slide 1
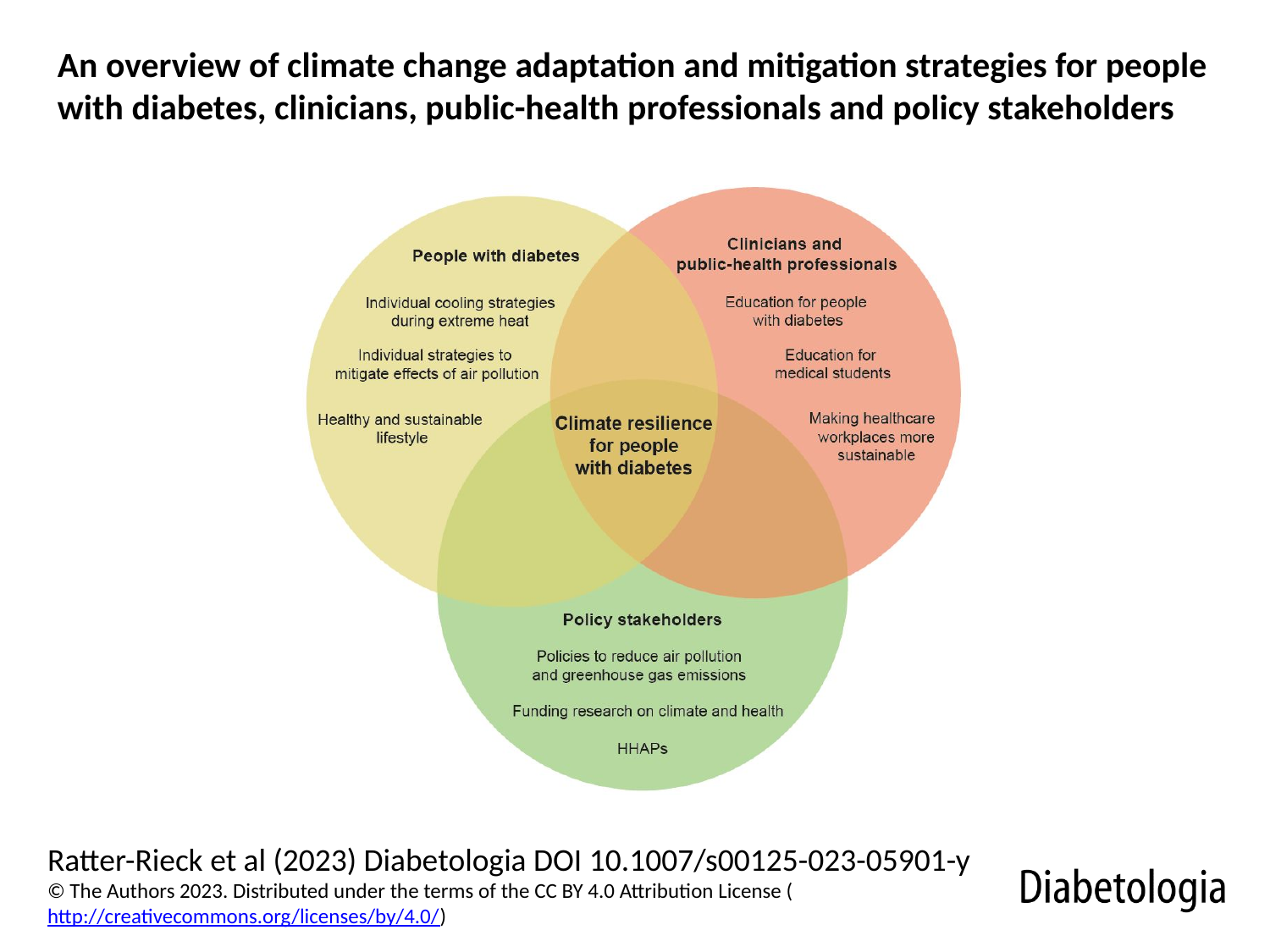

An overview of climate change adaptation and mitigation strategies for people with diabetes, clinicians, public-health professionals and policy stakeholders
Ratter-Rieck et al (2023) Diabetologia DOI 10.1007/s00125-023-05901-y
© The Authors 2023. Distributed under the terms of the CC BY 4.0 Attribution License (http://creativecommons.org/licenses/by/4.0/)

## Slide 2
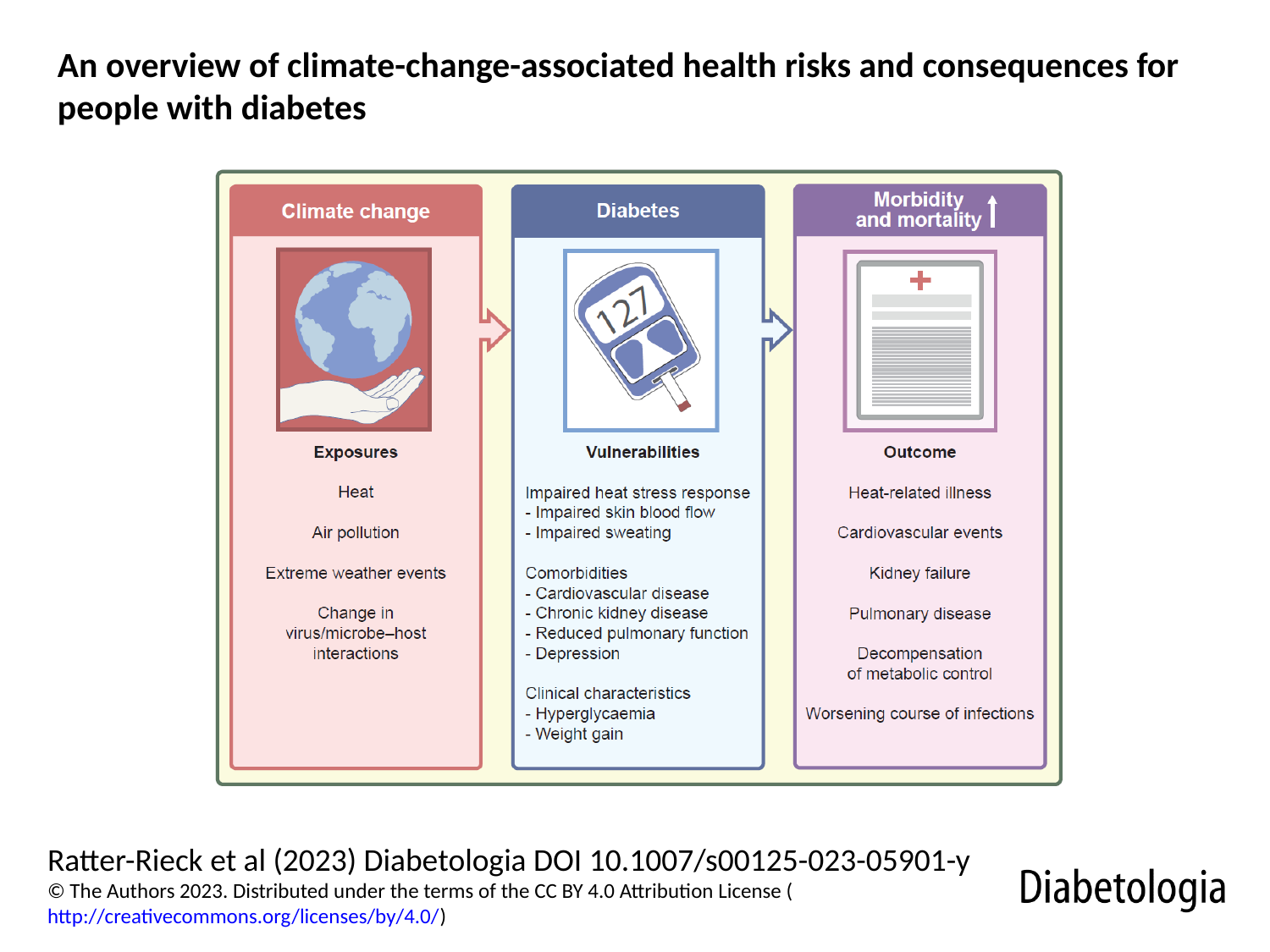

An overview of climate-change-associated health risks and consequences for people with diabetes
Ratter-Rieck et al (2023) Diabetologia DOI 10.1007/s00125-023-05901-y
© The Authors 2023. Distributed under the terms of the CC BY 4.0 Attribution License (http://creativecommons.org/licenses/by/4.0/)
